# Identification of novel TUBB1 variants in patients with macrothrombocytopenia

**DOI:** 10.3906/sag-2003-259

**Published:** 2021-04-30

**Authors:** Zihni Onur ÇALIŞKANER, Abdullah Abdul WAHEED, Merve TUZLAKOĞLU ÖZTÜRK, Yeşim OYMAK, Uygar Halis TAZEBAY, Nejat AKAR, Ayten KANDİLCİ, Didem TORUN ÖZKAN

**Affiliations:** 1 Department of Molecular Biology and Genetics, Faculty of Science, Gebze Technical University, Kocaeli Turkey; 2 Department of Bioengineering, Faculty of Engineering and Natural Sciences, Üsküdar University, İstanbul Turkey; 3 Department of Pediatric Hematology, Dr. Behçet Uz Children’s Hospital, İzmir Turkey; 4 Department of Pediatrics, Faculty of Medicine, TOBB-ETU University, Ankara Turkey; 5 Medical Laboratory, Vocational School of Health Services, İstanbul Okan University, İstanbul Turkey

**Keywords:** TUBB1, macrothrombocytopenia, MYH9, platelets

## Abstract

**Background/aim:**

Macrothrombocytopenia is an autosomal-dominant disorder characterized by increased platelet size and a decreased number of circulating platelets. The membrane skeleton and the link between actin filaments of the skeleton and microtubules, which consist of alpha and beta tubulin [including the tubulin beta-1 chain (TUBB1)] heterodimers, are important for normal platelet morphology, and defects in these systems are associated with macrothrombocytopenia.

**Materials and methods:**

In this study, we sequenced the exons of the *TUBB1* gene using DNA isolated from the peripheral blood samples of healthy controls (n = 47) and patients with macrothrombocytopenia (n = 37) from Turkey. The *TUBB1* expression levels in fractioned blood samples from patients and healthy controls were analyzed by RT-qPCR and Western blot. Microtubule organization of the platelets in the peripheral blood smears of patients, and in mutant *TUBB1* -transfected HeLa cells, were analyzed by immunofluorescence staining.

**Results:**

A new *TUBB1* c.803G>T (p.T178T) variant was detected in all of the control and patient samples. Importantly, we found 3 new heterozygous *TUBB1* variants predicting amino acid substitutions: G146R (in 1 patient), E123Q (in 1 patient), and T274M (in 4 patients); the latter variant was associated with milder thrombocytopenia in cancer patients treated with paclitaxel. Ectopic expression of TUBB1 T274M/R307H variant in HeLa cells resulted in irregular microtubule organization.

**Conclusion:**

Further clinical and functional studies of the newly identified TUBB1 variants may offer important insights into their pathogenicity in macrothrombocytopenia.

## 1. Introduction

Although the frequency of rare bleeding disorders ranges from 1 in 500,000 to 1 in 2 million, in countries with a high rate of consanguineous marriages they occur more frequently and constitute an important health problem [1]. Macrothrombocytopenia is a congenital autosomal-dominant blood disorder characterized by increased platelet size and a decreased number of circulating platelets [2]. Macrothrombocytopenia is classified within the genetically heterogeneous group of rare disorders. There are more than 12 genes that are thought to be associated with macrothrombocytopenia including *MYH9 (MIM155100)* , *ACTN1 (MIM615193) GP1A* (MIM606672), *GP9* (MIM173515), *FL1* (MIM193067), *FLNA* (MIM3000017), *ITG2A* / *ITGB3* (MIM 607759/173470), *GATA1* (MIM305371), *NBEAL2* (MIM614169), and *VWF* (MIM613160). Although the mutations in these genes have been associated with macrothrombocytopenia, an understanding of their role in the development of the disease remains elusive [3,4].

The membrane skeleton, and the link between actin filaments of the skeleton and microtubules, maintain normal platelet morphology [5], which is critical for macrothrombocytopenia given that patients display defects in the size and structure of platelets. Alpha- and beta-tubulin heterodimers are the key components of these structures [6]. Among the beta-tubulin isoforms, expression of *TUBB1* is restricted to the megakaryocytes and platelets, and its expression is induced during the differentiation of megakaryocytes, suggesting a role in proplatelet production and platelet release [7]. *TUBB1* , a 7401 base-pair gene, is located on human chromosome 20q13.3 and consists of 4 exons which encode 451 amino acids [6]. It functions in the building of the marginal band in platelets, a unique cytoskeleton structure that is composed of bundles of circumferential microtubules that support the maintenance of the shape and function of platelets [8,9]. Sequencing studies in patients with macrothrombocytopenia described several *TUBB1* variants that are mainly found in exon 4, which is the longest exon of the gene. Whether the same variants exist with similar frequency and whether there are additional undescribed *TUBB1* variants in Turkey is not known. Here, we sequenced the *TUBB1* gene in patients with macrothrombocytopenia and in healthy controls from Turkey using exon-specific PCR. Due to sample limitations, we were only able to analyze *TUBB1* mRNA expression and platelet structure in a sample of one patient carrying the missense variants of *TUBB1* (p.T274M/p.R307H). We also generated a Flag-tagged TUBB1 variant (p.T274M/p.R307H) by using the cDNA of the same patient. The microtubule structure of HeLa cells expressing this ectopic Flag-tagged mutant TUBB1 was analyzed using confocal microscopy after IC staining with ACTIN and TUBB1 (or Flag) antibodies.

## 2. Materials and methods

### 2.1. Blood sampling, DNA isolation, and sequencing

Written, informed consent for genetic analysis was obtained from the patients. Approval was also obtained from the Okan University Clinical Research Ethics Committee (24.09.2014; no: 09.2014.50) in order to comply with the ethical rules of the study. Blood samples of the patients (n = 37) and healthy controls (n = 47) were obtained from various hematology clinics in Turkey. Platelet counts of all patients and healthy controls were evaluated. Patients were asymptomatic at the time of admission, and peripheral blood sampling revealed macrothrombocytopenia. Platelet counts of 150–450 × 103/uL and MPV values of 7.5 fL–11.5 fL were accepted as control in the study. These were then subjected to molecular genetic analysis. The MYH9-related disorder was previously thought to be four separate disorders; May–Hegglin anomaly, Epstein syndrome, Fechtner syndrome, and Sebastian syndrome. All of these disorders involved thrombocytopenia and enlarged platelets and were distinguished by some combination of hearing loss, renal disease, and cataracts. Nonhematological findings (renal disease, cataracts, hearing loss, etc.) were evaluated by the relevant clinics.

The PCR reactions were performed using 25 ng DNA and GoTaq green master mix reagent in a 20 µL PCR volume (Promega, Wisconsin, USA). Primers were designed using an online tool from the Santa Cruz Genome Browser Santa Cruz Genomic Institute. Genome Browser. Website https://genome.ucsc.edu/ (Table 1). Sequences were analyzed with the Finch TV program.

**Table 1:** Primers that were used in the PCR reactions and sequencing of TUBB1.

Exons	Forward primer	Reverse primer	Amplicon(bp)
1	CATACCACGGTCACTAGGGC	AAAGCCCAAAGGCATTGTC	587
2	GGAAACAGGCTTGGGAATG	CATTTCCATCTCCTGGGC	282
3	TTTGGACCAGTATCACAAAGTTC	AAAAGAGAAACCAGCAGGGG	295
4 – 1	GCATTCGATCTAGCAAATTAGG	ATCGAACATCTGCTGGGTG	766
4 – 2	TACCCTGAAGCTGACGACAC	GCTGCAGGAGAAACACTCATC	789
4 – 3	AACGAATTTGGGGAAGCTG	GAAAGCAGGATGCCAGTCAG	608
4 – 4	GGATTTGCAGGGAGCCAC	TTCCTGCATTTGAATGGTTG	623
4 – 5	TTTCATTCAGTCATCACCCC	TTGGTATGTATTTTAGTTATTTCCTCG	604
4 – 6	GGGTTCTCATCTCTTGATTTGC	CGAAAGAGTAGGATGGTGAGATG	620

### 2.2. RNA isolation and RT-qPCR

Platelet-rich plasma (PRP) and peripheral blood mononuclear cells (PBMC) were isolated from the sample of patient #7 (Tables 2 and 3), who carries heterozygous *TUBB1* variants (p.T274M/p.R307H) in *cis* -position, and healthy volunteers (n = 4; pooled samples) using Lymphoprep density gradient media (Axis–Shield, Dundee, UK), according to manufacturer instructions. Total RNA was extracted using TriPure isolation reagent (Roche, Penzberg, Germany), and 1 µg of RNA was reverse transcribed to cDNA using the High-Capacity cDNA reverse transcription kit (Applied Biosytems, California, USA), following manufacturer instructions. The *TUBB1* and *ACTB* (housekeeping gene) mRNA levels in the PRP and PBMC of the patient and control samples were analyzed by RT-qPCR using Taqman primer-probe sets in triplicate ( *TUBB1* hs00258236_m1; ACTIN hs01060665_g1) (Applied Biosystems, California, USA). The ΔΔCt method was used to calculate relative gene expression.

**Table 2:** The summary of variants found in exon-4 of TUBB1 gene in Turkish population.

Exon-4 specificprimer pairs	Alteration type	Amino acidchange	Nucleotide change
Exon 4.1	Silent	T178T	c. 803G > TAll Patients (n=37) and controls (n=47)
Exon 4.1	Missense	G146R	c.705 G > APatient #36
Exon 4.1	Missense	E123Q	c.636 C > G Patient #17
Exon 4.1	Silent	H122H	635 C > TPatient # 30
Exon 4.2	Missense	T274M	821 C > TPatients #2,3,7,37
Exon 4.2	Known polymorphism in macrotrombocytopenia	R307H	920 G > A19 out of 37 Patients 13 out of 47 controls

**Table 3:** Results of MYH9 and TUBB1 genomic DNA analysis.

ID	Sex	Clinical diagnosis	MYH9 Variants		TUBB1 Variants		
			Found only inpatients	Found in controls and patients	Found only in patients	Found in controls andpatients	ACMGclassification
1	M	Macrothrombocytopenia	-	-	-	c.803 G>T, c.920 G>A	
2	M	Macrothrombocytopenia	c.3756 C>A,c.3762 G>A	-	c.821 C>T	c.803 G>T, c.920 G>A	PM2/PS3
3	F	Macrothrombocytopenia	-	-	c.821 C>T	c.803 G>T	PM2/PS3
4	M	Macrothrombocytopenia	-	c. 3660 T>G	-	c.803 G>T	
5	M	Macrothrombocytopenia	-	-	-	c.803 G>T, c.920 G>A	
6	M	Thrombocytopenia	c. 197 G>C	-	-	c.803 G>T, c.920 G>A	
7	F	Macrothrombocytopenia	-	c. 3660 T>G	c.821 C>T	c.803 G>T, c.920 G>A	PM2/PS3
8	F	May Hegglin Anomaly	-	-	-	c.803 G>T	
9	M	Macrothrombocytopenia	-	-	-	c.803 G>T	
10	M	Thrombocytopenia	-	-	-	c.803 G>T	
11	M	Macrothrombocytopenia	-	-	-	c.803 G>T, c.920 G>A	
12	F	Thrombocytopenia	-	-	-	c.803 G>T, c.920 G>A	
13	M	Macrothrombocytopenia	-	-	-	c.803 G>T, c.920 G>A	
14	M	Macrothrombocytopenia	-	-	-	c.803 G>T, c.920 G>A	
15	M	Thrombocytopenia	-	-	-	c.803 G>T	
16	M	Macrothrombocytopenia	c. 286 T>G	-	-	c.803 G>T	
17	M	Macrothrombocytopenia	-	-	c.636 C>G	c.803 G>T, c.920 G>A	PM2/PS1
18	M	May Hegglin Anomaly	-	-	-	c.803 G>T, c.920 G>A	
19	M	Macrothrombocytopenia	c. 3814 T>G	-	-	c.803 G>T	
20	M	Macrothrombocytopenia	-	-	-	c.803 G>T	
21	F	Thrombocytopenia	-	-	-	c.803 G>T	
22	M	Gray Platelet Syndrome	-	-	-	c.803 G>T, c.920 G>A	
23	M	Thrombocytopenia	-	-	-	c.803 G>T	
24	M	Thrombocytopenia	-	-	-	c.803 G>T, c.920 G>A	
25	M	Macrothrombocytopenia	-	-	-	c.803 G>T	
26	M	Macrothrombocytopenia	-	-	-	c.803 G>T	
27	F	Macrothrombocytopenia	-	-	-	c.803 G>T, c.920 G>A	
28	M	Macrothrombocytopenia	-	c. 3660 T>G	-	c.803 G>T	
29	M	Macrothrombocytopenia	-	-	-	c.803 G>T, c.920 G>A	
30	F	Macrothrombocytopenia	-	-	c.635 C>T	c.803 G>T, c.920 G>A	PM2/PS1
31	F	Thrombocytopenia	-	-	-	c.803 G>T	
32	M	Macrothrombocytopenia	-	-	-	c.803 G>T	
33	F	Thrombocytopenia	-	-	-	c.803 G>T, c.920 G>A	
34	M	May Hegglin Anomaly	-	-	-	c.803 G>T	
35	M	Thrombocytopenia	-	-	-	c.803 G>T	
36	M	Thrombocytopenia	-	-	c.705 G>A	c.803 G>T, c.920 G>A	PM2/PS1
37	M	Thrombocytopenia	-	-	c.821 C>T	c.803 G>T, c.920 G>A	PM2/PS3

### 2.3. TUBB1 cloning and transfection

The C-terminus Flag-tagged wild type and mutant (p.T274M/p.R307H) full-length *TUBB1* cDNA was synthesized from the PBMC of patient #7 and cloned into p3XFLAG-CMV-14 expression vector (Sigma–Aldrich, Missouri, USA) using the following primers: forward 5’-TACGGCGGCCGCGATGCGTGAAATTGTCCATAT

TCA-3’ and reverse 5’-TACGGGTACCATGTCCCTTATC

TTCTGG-3’. The integrity of the constructs was confirmed by sequencing. HeLa cells were transiently transfected with these vectors using jetPRIME transfection reagent (Polyplus Transfection, Illkirch, France), following manufacturer instructions.

### 2.4. Immunofluorescence staining

For immunofluorescence (IF) staining, peripheral blood smears or the transfected HeLa cells that were seeded on the chamber slides were fixed with absolute methanol at –20 °C for 15 min. After washing with PBS-T (0.1% Triton-X-100), slides were incubated in blocking/permeabilization solution (2% skim milk in PBS-T) at room temperature for 1 h. Transfected HeLa cells were incubated with mouse antihuman α-TUBULIN antibody (Santa Cruz, sc-5286; 1:100 dilution, Texas, USA) and rabbit antihuman Flag antibody (Sigma–Aldrich, F7425; 1:100 dilution). Slides of fixed blood smears were incubated with mouse antihuman TUBB1 antibody (OriGene, TA506654, 1:150 dilution, Maryland, USA) and rabbit antihuman α-TUBULIN antibody (Cell Signaling, 2125; 1:100 dilution, Massachusetts, USA). After washing, slides were incubated with corresponding anti-IgG antibodies with Alexa-488 (Cell Signaling, 4412; 1:500 dilution) or Alexa-555 fluorophore (Cell Signaling, 4409; 1:500 dilution) at room temperature for 1 h. Slides were mounted with DAPI solution (1 µg/10 mL prepared in ddH2O) for nuclei staining. Stained cells were visualized under LSM 880 laser scanning confocal microscope (Zeiss, Göttingen, Germany).

### 2.5. Western blot analysis

Whole protein lysate was isolated using RIPA buffer [150 mMNaCl, 50 mMTris-HCl (pH = 8), 1% NP40, 0.5% sodium deoxycholate, 0.1% SDS] completed with 100X protease/phosphatase inhibitor cocktail (Thermo Fisher, Massachusetts, USA), and protein concentration was measured by BCA protein assay kit (Thermo Fisher). Each lysate from different samples was separated by using Mini-PROTEAN TGX 10% precast gel (Bio-Rad, California, USA) and transferred to nitrocellulose membrane using Trans-Blot Turbo transfer system (Bio-Rad). Membranes were blocked with 5% BSA (Sigma–Aldrich) in TBS-T and then incubated with a mouse antihuman TUBB1 antibody (OriGene, TA506654; 1:3000 dilution) at 4 °C overnight, an anti-Flag HRP antibody (Abcam, ab49763; 1:1000 dilution, Cambridge, UK) at room temperature for 1 h, a rabbit antihuman GAPDH antibody (Cell Signaling, 5174; 3:10000 dilution) at room temperature for 1 h, or a rabbit antihuman β-actin antibody (Cell Signaling, 8457; 1:1000 dilution) at room temperature for 1 h. Upon washing with TBS-T, membranes were incubated with compatible HRP-conjugated antimouse IgG (Cell Signaling, 7076; 1:5000 dilution) or antirabbit IgG (Cell Signaling, 7074; 1:5000 dilution) secondary antibodies at room temperature for 1 h. Protein bands were detected using ECL Plus Western blotting substrate (Thermo Fisher) and ChemiDoc XRS+ imaging system (Bio-Rad).

### 2.6. Bioinformatic analysis

Computational mutation analysis was performed using online prediction tools such as PROVEAN http://provean.jcvi.org, MutationTaster2 http://www.mutationtaster.org, and PolyPhen-2 http://genetics.bwh.harvard.edu/pph2/. MutationTaster2 was also used for the multiple amino acid sequence alignment of *TUBB1* homologues.

## 3. Results

### 3.1. Sequencing of TUBB1 exons

**Figure 1:**
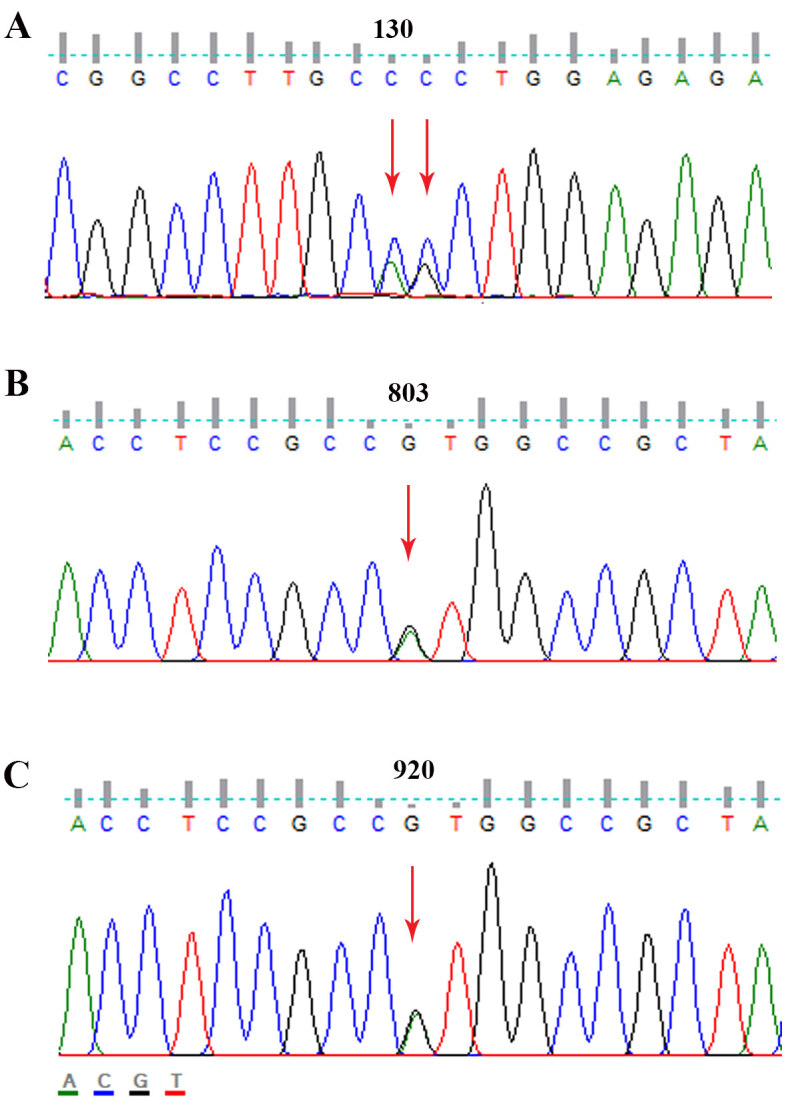
Entire coding exons were amplified by PCR and sequenced. Heterozygous variations (A) c.130-131AG>CC (p.Q43P), (B) c.803G>T (p.T178T), and (C) c.920G>A (p.R307H) were detected on TUBB1 gene from healthy controls and/or macrothrombocytopenia patients by FinchTV sequence analysis software.

**Figure 2:**
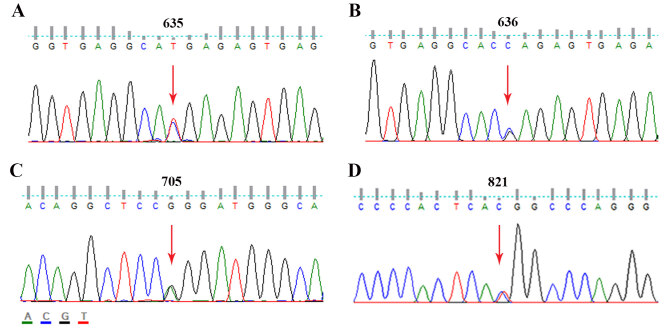
Sequencing also revealed heterozygous TUBB1 variations identified for the first time in macrothromboytopenia patients in Turkish population. (A) A silent TUBB1 variation c.635C>T (p.H122H), and 3 missense variants (B) c.636C>G (p.E123Q), (C) c.705G>A (p.G146R), and (D) c.821C>T (p.T274M) were detected in patient #30, patient #17, patient #36 and patients #2, #3, #7, and #37, respectively.

**Figure 3:**
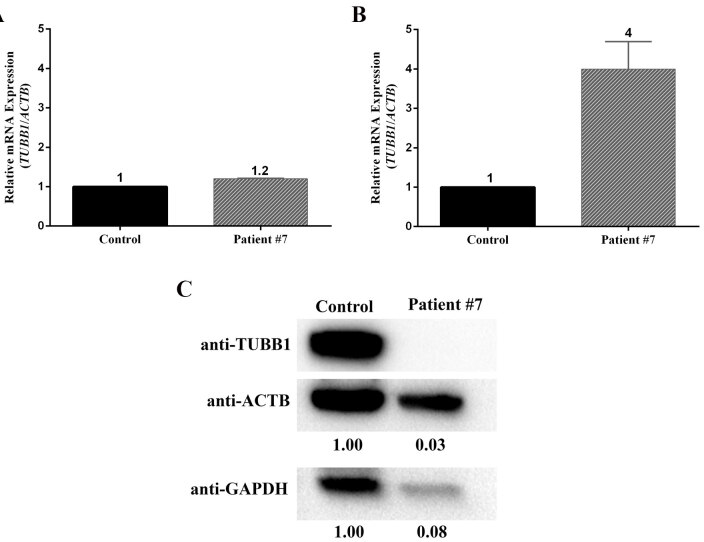
TUBB1 expression was detected in both mRNA and protein levels by using blood samples from healthy control (n=4, pooled) and patient #7. RT-qPCR analysis showed relative TUBB1 gene expression in both (A) PBMC and (B) PRP fractions. All Taqman assays were carried out in triplicates and relative fold change was calculated with the ΔΔCt method. (C) TUBB1 protein band was not detectable in the whole lysate isolated from patient #7 contrary to healthy control.

Initially, we sequenced all of the exons (1 to 4) of *TUBB1* in patient samples (n = 37) to determine any possible *TUBB1* variant. Healthy controls (n = 47) were subsequently sequenced to screen for *TUBB1* variants that we found in patient samples. All of the determined variants were confirmed by repeating the corresponding PCR reactions and using forward and reverse primers in the sequencing analysis. Sequencing results of the patient samples did not show any new variant in exons 1, 2, or 3 of *TUBB1* ; however, in exon-2 we found heterozygous *TUBB1* variant c.130-131 AG>CC (p.Q43P) in 4 patients (11%) and 7 healthy controls (15%) (Figure 1A), which had been previously reported as a functional polymorphism in congenital macrothrombocytopenia patients [10]. Given that the fourth exon of *TUBB1* is the largest one (2950 base pairs) and contains most of the published genetic variations [6], we designed 6 different primer pairs to sequence this exon (Table 1). Primer pairs, named exon 4.1 and 4.2 (Table 1), cover the coding region of the 4th exon. The *TUBB1* variants described below were all covered within the PCR products of the 4.1 and 4.2 primer pairs. In this region of the 4th exon, we found a novel synonymous *TUBB1* c.803G>T (p.T178T) variant in all of the healthy control and patient samples (Figure 1B) (Table 3). In addition, we showed previously-described *TUBB1* variant c.920 G>A (p.R307H) [11,12] for the first time in patients (19 out of 37; 51%) and healthy controls (13 out of 47; 28%) from Turkey (Figure 1C) (Table 3). Importantly, we identified one new silent ( *TUBB1* c.635C>T; p.H122H in patient #30) (Figure 2A) and 3 new heterozygous missense *TUBB1* variants in patient samples only (c.636C>G, p.E123Q in patient #17; c.705G>A p.G146R in patient #36; and c.821C>T p.T274M in patients #2, #3, #7, and #37) (Tables 2 and 3) (Figures 2B–2D). In the literature, the TUBB1 variant T274M was associated with milder thrombocytopenia in cancer patients treated with paclitaxel [13]; however, to our knowledge, it was not previously described in macrothrombocytopenia patients. The patients carrying p.G146R, p.E123Q, and T274M were also positive for the R307H variant. Two of the T274M carriers (Table 3; patients #2 and #3) were relatives (a son and his mother, respectively). We also showed that patients #2 and #7 are carriers of *TUBB1* variant c.920G>A (p.R307H) and *MYH9* variants c.3756C>A (p.L1176M)/ c.3762G>A (p.E1182K) and c.3660T>G (p.A1144L), respectively (Table 3); this is the first presentation of the coexistence of the *TUBB1* and *MYH9* variants in macrothrombocytopenia patients. Since we did not find *TUBB1* variation in the remaining portions of exon 4 in the patient samples (screened with exon 4.3, 4.4, 4.5, and 4.6 primers), we did not screen these regions of exon 4 in healthy control samples. 

###  T274M/R307H changes the microtubule structure 3.2. TUBB1 variant p. T274M/R307H changes the microtubule structure

**Figure 4:**
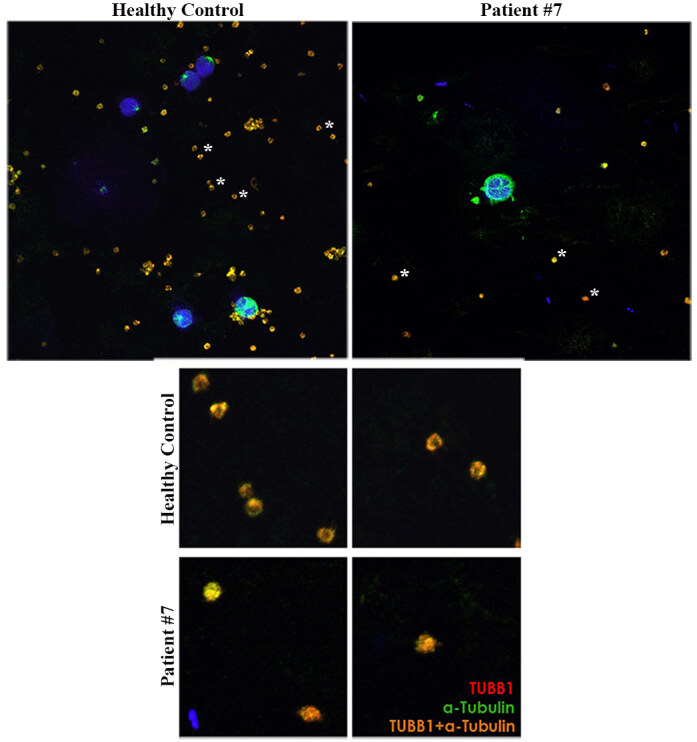
Peripheral blood smears were stained for marginal band detection in healthy or patient’s platelets. Endogenous TUBB1 and α-Tubulin which are subunits of marginal band were marked with specific primary antibodies in combination with Alexa-555 (red) or Alexa-488-conjugated (green) secondary antibodies, respectively. Marginal band was determined as a hollow-ring structure in health platelets but circular microtubule arrangement was disappeared in patient #7’s platelets carrying TUBB1 variant p.T274M/R307H. Merged images (yellowish) were captured in oiled 63X magnification by using LSM 880 (Zeiss) confocal microscope (upper panel). Platelets labeled with an asterisk were zoomed in while image processing (bottom panel).

**Figure 5:**
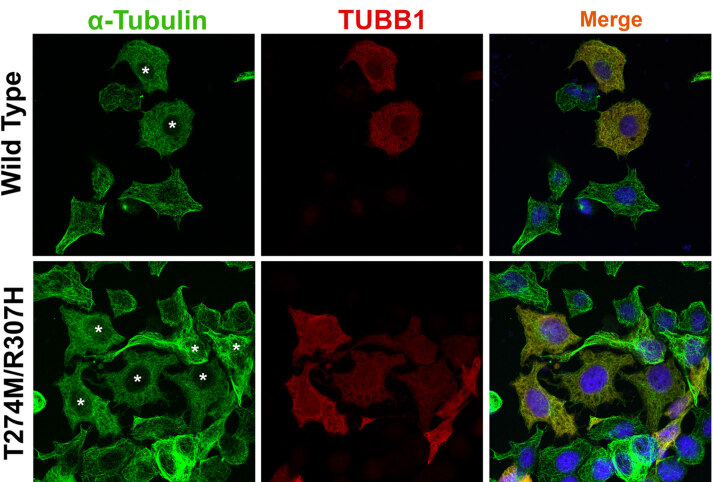
IF analysis of HeLa cells transiently transfected with C-terminus Flag-tagged wild-type TUBB1 (WT) or T274M/R307H variant constructs. Exogenous TUBB1 was labeled with anti-Flag primary antibody (Sigma-Aldrich, F7425; 1:100 dilution) and α-Tubulin was labeled with anti-alpha Tubulin primary antibody (Santa Cruze, sc-5286; 1:100 dilution) in combination with Alexa-555 (red) or Alexa-488-conjugated (green) secondary antibodies, respectively. Cells expressing WT protein have a branched microtubule organization but transfection of T274M/R307H variant resulted in a disrupted and diffuse microtubule staining compared with WT and untransfected cells. Successfully transfected and exogenous Flag-tagged TUBB1 expressing HeLa cells were labeled with an asterisk. Images were captured in oiled 63X magnification by using LSM 880 (Zeiss) confocal microscope.

Due to sample limitations and the unavailability of other patients carrying heterozygous missense mutations (p.G146R and p.E123Q), we were able to clone and analyze only the effect of TUBB1 variant p.T274M/R307H (rs35565630/rs6070697) on the morphology of the platelets of patient #7 and on the microtubule organization of HeLa cells. The clinical data of patient #7 is summarized in Table 4. Both variants exist in the ExAC database with high MAF values (p.T274M MAF value: 0.018 and p.R307H MAF value: 0.173). Computational mutation analysis tools PROVEAN, MutationTaster2, and PolyPhen-2 also predicted deleterious effects of p.T274M variation on TUBB1 protein function (data not shown). Endogenous *TUBB1* mRNA was detectable in both the peripheral blood mononuclear cells (PBMC) and platelet-rich plasma samples of patient #7 (Figures 3A and 3B), who is heterozygous for the variant p.T274M/R307H. Although the endogenous TUBB1 protein was detectable in the platelet samples of a healthy control, we could not detect the TUBB1 protein in the platelets of patient #7 using Western blot analysis (Figure 3C). The IC analysis of platelets by confocal microscopy showed a normal marginal band structure (ring shaped) in a healthy control sample; however, the marginal band was disrupted and a diffuse staining with both α-TUBULIN and TUBB1 antibodies was observed in the majority of platelets from the same patient (Figure 4). We also transiently expressed the Flag-tagged TUBB1 variant p.T274M/R307H in the HeLa cell line to determine its effect on microtubule distribution under steady-state conditions. The IC analysis of HeLa cells transfected with TUBB1 p.T274M/R307H variant showed relatively diffuse cytoplasmic staining with anti α-TUBULIN and anti-Flag antibodies (labels the Flag-tagged TUBB1), whereas the control HeLa cells transfected with wild type Flag-tagged TUBB1 showed a more organized and clear microtubule net. These results suggested that TUBB1-p.T274M/R307H variant causes a disorganized microtubule structure under steady-state conditions (Figure 5). 

**Table 4:** Clinical data of all patients carrying newly identified TUBB1 variants and patient #7 which is proceeded for functional analysis.

Case	#2	#3	#7	#17	#30	#36
Age	21	13	13	12	4	14
Sex	Male	Male	Female	Male	Male	Female
WBC (103/ uL)	14.47	3.9	11.4	6.68	10.57	7.9
RBC (uL)	4.88	4.49	4.38	5.23	4.9	4.57
HB (g / dL )	14.3	11.9	11.4	13.6	11.6	12
HCT (%)	42.3	35	38.1	40.3	36.2	36.1
MCV (fL)	86.7	78.1	78.3	77.1	73.9	79.1
MCH (pg)	29.3	26.6	26.2	26	23.7	26.3
MCHC (g/dL)	33.8	34.1	32.8	33.7	32	33.3
PLT (103/ uL)	52	89	32	29	87	69
MPV (fL)	9.9	8.4	11.5	nd	13	9.6
PDW (fL)	12.2	17.1	13.6	nd	18.1	16
PCT (%)	0.05	0.074	0.04	nd	0.11	0.31
Consanguineous marriages	-	+	+	-	-	-
Bleeding problem	+	-	-	-	-	-
Renal disease	-	-	-	+	-	-
Cataracts	-	-	-	-	-	-
Hearing loss	-	-	-	-	-	-

Interestingly, we also found the MYH9 (p.K64N) and TUBB1 (p.R307H) variants in the same patient (patient #7). While the variant in MYH9 causes a defect in proplatelet formation and platelet secretion [14,15], an understanding of the effects of the coexistence of these two variants remains elusive.

## 4. Discussion

In the current study we screened all exons of the *TUBB1* gene using DNA from the peripheral blood samples of patients with macrothrombocytopenia from Turkey (n = 37) (Table 3). The patients were selected based on symptoms affecting platelet morphology, such as large abnormal platelet structure, and the platelet count. We also included DNA samples from healthy controls (n = 47). 

We found 2 types of previously described TUBB1 polymorphisms (Q43P and R307H) for the first time in Turkey. We also showed 3 new missense and 1 new silent variant in the fourth exon of *TUBB1* in the macrothrombocytopenia patients but not in healthy controls (Table 2), which suggests that these are not common polymorphisms. The findings are of great importance, as they represent single nucleic acid base changes which affect the amino acid sequences in the protein. These changes can end up providing an abnormal biochemical property, such as charge, hydrophobicity, or size, within the wild type protein. To check this theory, different function prediction programs such as PROVEAN and Poly Phen-2 were utilized. Both analyses suggest that the variants G146R and T274M have potentially deleterious effects on TUBB1 (data not shown). The p.G146R (the glycine in position 146 of β1‐tubulin) is highly conserved, and the variation is located in the amino‐terminal guanosine triphosphate domain of β1‐ tubulin. In silico modeling confirmed its strong influence on the structure of this domain, and the gene where this mutation occurs is related to macrothrombocytopenia.

Reviewing TUBB1 and its genetic variations in macrothrombocytopenia to date, we found that reported mutations in mammals included 2 missense mutations, p.R318W and p.F260S in exon 4 in humans [8,16], and a missense mutation of p.D249N in canines [17]. All of these variations are believed to be located in or near the α- and β-tubulin intradimer interface, reportedly causing macrothrombocytopenia in humans and dogs, respectively. The intradimer interface of the platelet structure is believed to be disrupted by these mutations, causing frayed cytoskeleton formation and affecting platelet morphology. With the exception of p.R307H and p.Q43P polymorphisms, the variants discovered in macrothrombocytopenia patients in our study are distinct and do not overlap with earlier studies performed in different populations, which suggests an evolutionary difference in the Turkish population. Previously it was shown that the ectopic expression of R307H variant in human MCF7 cells causes defects in microtubule regrowth after treatment of the cells with nocodazole, a reagent that depolymerizes the microtubules [18]. Given that it is observed at high frequency in healthy individuals [27% in our study (Tables 2 and 3) and 30% in Basciano et al.] [18], it is likely that its effect on platelets is not disastrous. On the other hand, although the p.T274M/R307H variant was seen at a low frequency (0.023) in Caucasian populations without a known history of macrothrombocytopenia, we describe for the first time a tendency towards this variant in macrothrombocytopenia patients (4 out of 37; 10%), but not in healthy individuals from Turkey (n = 47); this suggests a potential role for variant T274M/R307H in the disturbed platelet structure that we observed in the platelets of the patient sample (Figure 4). In support of our hypothesis, Western blot analysis demonstrated that MCF7 cells express ectopic p.T274M or p.T274M/R307H, and cancer patients carrying these variants are less sensitive to the polymerization effect of paclitaxel on microtubules [13]. Supporting these findings, we showed for the first time that cytoplasmic distribution of the microtubule net was disturbed under steady-state conditions in HeLa cells.

Although we were not able to functionally link the variants p.G146R and p.E123Q to macrothrombocytopenia, our data involving the T274M/R307H variant suggest that it may be useful as a biomarker in immune thrombocytopenia given its potential effect on platelet turnover (which leads to lower platelet counts). Finally, given that the synonymous TUBB1 p.T178T variant was found in all of our healthy control and patient samples, it is unlikely that this variant is associated with macrothrombocytopenia.

The American College of Medical Genetics and Genomics (ACMG) has recommended a five-tier classification system [19]. According to this classification system, a sequence change can be categorized as pathogenic, likely pathogenic, uncertain significance, likely benign, and benign. In our study, the p.705G>A, p.636C>G, and p.821C>T variants were found only in patients, and they had a small minor allele frequency (MAF) value, suggesting their pathogenicity [nsv2778234 (ClinVar)]. Additionally, the variants that we found in 6 patients (#2, #3, #7, #17, #30, and #36) could be categorized as PM2/PS3 (pathogenic modified/pathogenic strong criteria). Here, PM2 indicates that the mutation is found only in the patient group and is absent in the controls; PS3 shows the functional effect of mutation (Table 4).

In summary, our findings suggest that further functional and clinical analyses of the new TUBB1 variants that we identified in macrothrombocytopenia patients may provide new insights into the molecular mechanisms of normal and abnormal platelet production and morphology. 

## Author contributions

DTÖ and AK designed and guided the study, analyzed the data, and wrote the manuscript. ZOÇ and AAW performed the experiments and analyzed the data. MTÖ guided and helped the construction of the plasmids. NA and YO provided the patient material and clinical data. UHT guided the experiments and reviewed the data and manuscript.
